# Association Between Long-term Use of H_2_ Receptor Antagonists and Prostate Cancer Risk: A Case-Control Study in Taiwan

**DOI:** 10.7150/jca.125694

**Published:** 2026-01-14

**Authors:** Shao-Fu Wang, Yu-Chen Liu, Phung-Anh Nguyen, Guan-Ling Lin, Chih-Wei Huang, Annisa Ristya Rahmanti, Hsuan-Chia Yang

**Affiliations:** 1 School of Medicine, College of Medicine, Taipei Medical University, Taipei, Taiwan.; 2 Graduate Institute of Biomedical Informatics, College of Medical Science and Technology, Taipei Medical University, Taipei, Taiwan.; 3 School of Nursing, College of Medicine, National Taiwan University, Taipei, Taiwan.; 4 Clinical Big Data Research Center, Taipei Medical University Hospital, Taipei Medical University, Taipei, Taiwan.; 5 Graduate Institute of Data Science, College of Management, Taipei Medical University, Taipei, Taiwan.; 6 Clinical Data Center, Office of Data Science, Taipei Medical University, Taipei, Taiwan.; 7 Research Center of Health Care Industry Data Science, College of Management, Taipei Medical University, Taipei, Taiwan.; 8 Department of Health Policy and Management, Faculty of Medicine, Public Health and Nursing, Universitas Gadjah Mada, Yogyakarta, Indonesia.; 9 Department of Computer Science, Faculty of Science and Technology, Middlesex University, London, UK.; 10 International Center for Health Information Technology (ICHIT), Taipei Medical University, Taipei, Taiwan.; 11 Research Center of Big Data and Meta-analysis, Wanfang Hospital, Taipei Medical University, Taipei, Taiwan.

**Keywords:** histamine-2 receptor antagonists, prostate cancer, cancer risk, cimetidine, ranitidine

## Abstract

**Objective:** The association between long-term use of histamine-2 receptor antagonists and prostate cancer remains unclear. This study aimed to examine the age-specific risk of prostate cancer associated with long-term use of these medications.

**Methods:** We conducted a nationwide case-control study using Taiwan's Health and Welfare Data Science Center database from 2003 to 2016. Men with newly diagnosed prostate cancer were matched to controls, and long-term use was defined as cumulative exposure of sixty days or more. Adjusted odds ratios were estimated using conditional logistic regression, controlling for comorbidities and medications.

**Results:** Among 43,578 prostate cancer cases and 174,312 controls, long-term use of histamine-2 receptor antagonists was associated with a modest increase in prostate cancer risk, significant in men aged sixty-five and older (adjusted odds ratio = 1.087, 95% CI: 1.044-1.131) but not in younger groups. Cimetidine and ranitidine were each associated with increased risk in older men, while famotidine showed no significant association across age groups. Notably, cimetidine uses in men aged forty to sixty-four was associated with reduced prostate cancer risk (adjusted odds ratio = 0.865, 95% CI: 0.755-0.990), suggesting possible age-dependent effects.

**Conclusions:** These findings suggest that long-term use of cimetidine and ranitidine may increase prostate cancer risk in older men, while famotidine was not associated with prostate cancer risk. Risk varies by age and drug type, highlighting the need for drug-specific evaluation in cancer pharmacoepidemiology.

## 1. Introduction

Prostate cancer ranks fourth in incidence and eighth in mortality worldwide according to global cancer statistics in 2022 1, being the most frequently diagnosed cancer in men across 118 countries, with 1.47 million new cases and 396,792 deaths annually 2, 3. Androgen signaling plays a central role in prostate cancer development and progression, and age-related hormonal changes may influence individual susceptibility 4-6. While age, genetic predisposition, dietary habits, and environmental exposures are well-established risk factors 7-9, the possible role of long-term medication use in prostate cancer development has received relatively limited attention in epidemiological research.

Histamine-2 receptor antagonists (H₂RAs), including ranitidine, cimetidine, and famotidine, have been widely used to treat acid-related gastrointestinal disorders such as gastroesophageal reflux disease and peptic ulcers. Among them, cimetidine has been associated with an increased risk of prostate cancer in individuals who used it daily for 10 years when compared to non-users, while short-term use has shown no such association, possibly due to its inhibition of dihydrotestosterone binding to androgen receptors, increased plasma estradiol concentrations, and elevated prolactin levels, a potential growth factor for prostate cancer 10. Supporting this hypothesis, a 9-week high-dose cimetidine study in animals further suggested a potential link between cimetidine exposure and prostate carcinogenesis 11.

Notably, ranitidine has been the primary focus of cancer risk studies due to concerns regarding its contamination with N-nitrosodimethylamine (NDMA), a probable human carcinogen classified by the International Agency for Research on Cancer (IARC) 12-14. However, epidemiological studies on ranitidine use and cancer risk have reported inconsistent findings across different cancer types. A multinational cohort study using 12 international healthcare databases found no association between ranitidine use (≥30 cumulative days) and overall cancer incidence compared to other H₂RA users 15. Similarly, an analysis of medical data found no increased risk of overall or prostate cancer in ranitidine or nizatidine users compared to other H₂RA users, nor a significant dose-response relationship 16. In contrast, research indicated that individuals with at least 90 defined daily doses of ranitidine had a higher risk of liver, gastric, pancreatic, and lung cancers compared to non-users and famotidine users 17. Additionally, an analysis of bladder cancer risk found an increased incidence among individuals with at least three years of continuous ranitidine use compared to non-users 18.

Despite growing interest in cancer risk, studies focusing specifically on prostate cancer concerning H₂RA use remain scarce. Although ranitidine has been withdrawn from the market, other H₂RAs remain in use. Understanding the overall impact of H₂RA use, including historical exposure, is important for evaluating potential long-term risks or preventive effects. Moreover, a key limitation in existing research is the lack of age-stratified analyses. Since prostate cancer risk increases with age, and older adults tend to use H₂RAs more frequently and for longer durations, their cumulative exposure may differ significantly from younger individuals. Thus, this study aimed to explore the association between the long-term use of H₂RAs and prostate cancer risk across different age groups.

## 2. Methods

### 2.1 Data Resources

This population-based case-control study utilized data from the Taiwan Health and Welfare Data Science Center (HWDC) databases managed by Taiwan's Ministry of Health and Welfare 19. The HWDC databases have been in operation since Taiwan's National Health Insurance program was implemented in 1995. Covering 99.9% of Taiwan's population, approximately 23 million individuals, these databases provide comprehensive healthcare information, including outpatient and inpatient records, cancer registry data, and other healthcare-related datasets. 20. This integrated database contains detailed information on demographic characteristics (e.g., age, gender) and medical history (e.g., comorbidities, medication use, and healthcare utilization). The cancer registry database includes data on cancer diagnoses, tumor-node-metastasis (TNM) classification, diagnosis dates, histopathological confirmation, and primary treatment modalities such as surgery, radiotherapy, and hormone therapy 19. This study was approved by the Taipei Medical University - Joint Institutional Review Board (TMU-JIRB), under approval number N202003069.

### 2.2 Study Population

The study population consisted of individuals diagnosed with prostate cancer and a matched control group. Prostate cancer cases were defined as those who received their first prostate cancer diagnosis, as recorded in the cancer registry database, between January 1, 2003, and December 31, 2016. Individuals were excluded from the study if they had a previous cancer diagnosis between January 1, 2001, and December 31, 2002, were younger than 20 years of age, had unknown sex, or had a diagnosis date that was inconsistent or could not be identified. To ensure that only newly diagnosed prostate cancer cases were included and to minimize the risk of misclassification due to pre-existing diagnoses, a two-year washout period (2001-2002) was implemented.

**Figure 1:**
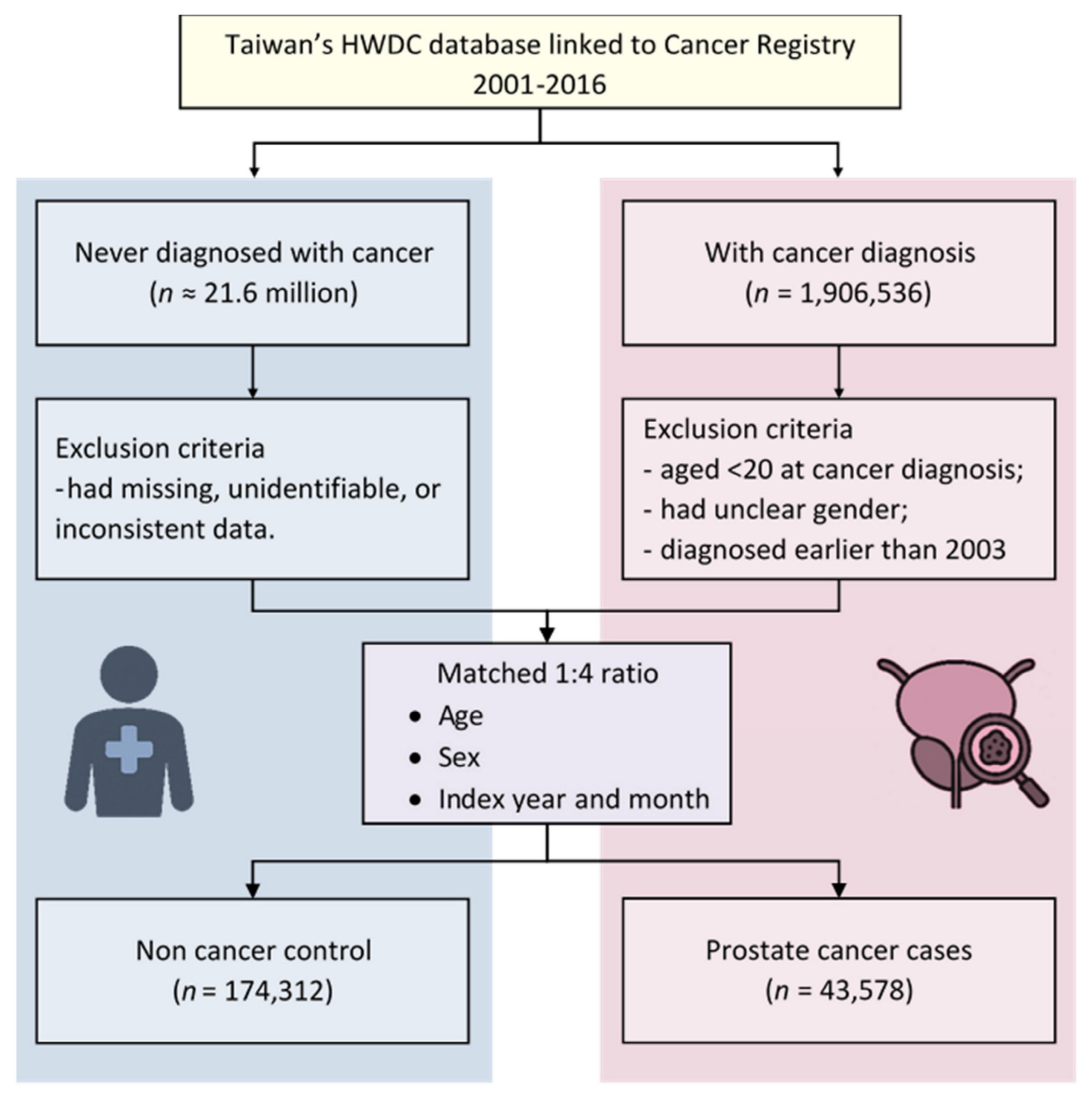
Flow diagram showing study design. HWDC, Health and Welfare Data Science Center.

Each prostate cancer case was matched with up to four controls based on age, sex, and the index year and month, using a 1:4 matching ratio. This approach aimed to enhance statistical power and reduce potential selection bias 21. A study flowchart outlining the methodology is presented in Figure 1.

### 2.3 Definition of H_2_RA Users

H₂RA users were identified based on outpatient and inpatient prescription records obtained from HWDC databases. Medications were classified using the World Health Organization Anatomical Therapeutic Chemical (ATC) classification system 22, specifically under the code A02BA for H₂RAs. The index date was defined as the year and month of the first prostate cancer diagnosis for cases, and the corresponding matched year and month for controls. Individuals were categorized as H₂RA users if they had a cumulative usage of ≥ 60 days prior to the index date. This definition mirrors established claims-based definitions for sustained use 23 and follows methodological standards to prevent exposure misclassification 24. Clinically, this cutoff distinguishes stable treatment implementation from incidental use 25.

Those who did not use any H₂RA or had cumulative use of less than 60 days were classified as non-users. For analyses of individual H₂RA drugs (cimetidine, ranitidine, and famotidine), users were identified independently for each medication, so that individuals could be classified as users of more than one H₂RA during the observation period.

### 2.4 Confounding Factor Adjustment

Potential confounders included comorbidities and medications associated with cancer risk. Comorbidities were assessed using diagnostic codes and summarized with the Charlson Comorbidity Index (CCI) 26, 27. Aspirin (ATC: B01AC06) 28 and metformin (ATC: A10BA02) 29, and statins (ATC: C10AA) 30 use were included due to their known associations with cancer.

### 2.5 Statistical Analysis

Conditional logistic regression was used to estimate odds ratios (ORs) and 95% confidence intervals (CIs) for the association between H₂RA use and prostate cancer risk. All models were adjusted for age-adjusted CCI scores and concomitant medications. Analyses were conducted overall and stratified by age groups (20-39, 40-64, and ≥ 65 years).

## 3. Results

### 3.1 Baseline Characteristics of Cases and Controls

A total of 43,578 prostate cancer cases and 174,312 matched controls were included in the study (Figure 1). The mean age in both groups was approximately 71 years, with individuals aged 65 years and older comprising 79.22% of the study population (Table 1).

Compared to controls, cases had higher prevalence of peptic ulcer disease, chronic pulmonary disease, mild liver disease, renal disease, and rheumatic disease, at 24.52%, 17.12%, 9.97%, 8.19%, and 1.06%, respectively. Regarding concomitant medications, the use of aspirin and metformin was more common in the case group compared to the control group.

### 3.2 Overall Association between H_2_RA Use and Prostate Cancer

In Table 2, long-term use of H₂RAs (≥ 60 cumulative days) was associated with a modest but statistically significant increase in prostate cancer risk compared to non-users (adjusted odds ratio [aOR] = 1.069; 95% confidence interval [CI]: 1.031-1.109; p = .0004).

In age-stratified analysis, this association remained significant among individuals aged 65 years and older (aOR = 1.087; 95% CI: 1.044-1.131; p < .0001), but was not observed in the 40-64 age group (aOR = 0.988; 95% CI: 0.898-1.087; p = .8012).

### 3.3 Drug-Specific Risk Estimates

When examining individual drugs, both cimetidine and ranitidine were associated with increased prostate cancer risk in the overall population. The adjusted odds ratio for cimetidine was 1.051 (95% CI: 1.002-1.101; p < .05), and for ranitidine was 1.131 (95% CI: 1.042-1.228; p < .01). In contrast, famotidine use was not significantly associated with prostate cancer risk (aOR = 1.016; 95% CI: 0.947-1.090; p >.05).

### 3.4 Age-Stratified Analysis of Individual H₂RA Drugs

Among individuals aged 65 years and older, both cimetidine and ranitidine users showed a significantly increased risk of prostate cancer. The adjusted odds ratios were 1.079 (95% CI: 1.026-1.135; p =.003) for cimetidine and 1.141 (95% CI: 1.044-1.248; p =.0037) for ranitidine.

In contrast, among individuals aged 40 to 64 years, cimetidine use was associated with a statistically significant reduction in prostate cancer risk (aOR =0.865; 95% CI: 0.755-0.990; p =.0358). Ranitidine and famotidine were not significantly associated with risk in this age group. Due to data de-identification, analysis for the 20-39 age group was not possible.

### 3.5 Summary of Risk Estimates

**Figure 2:**
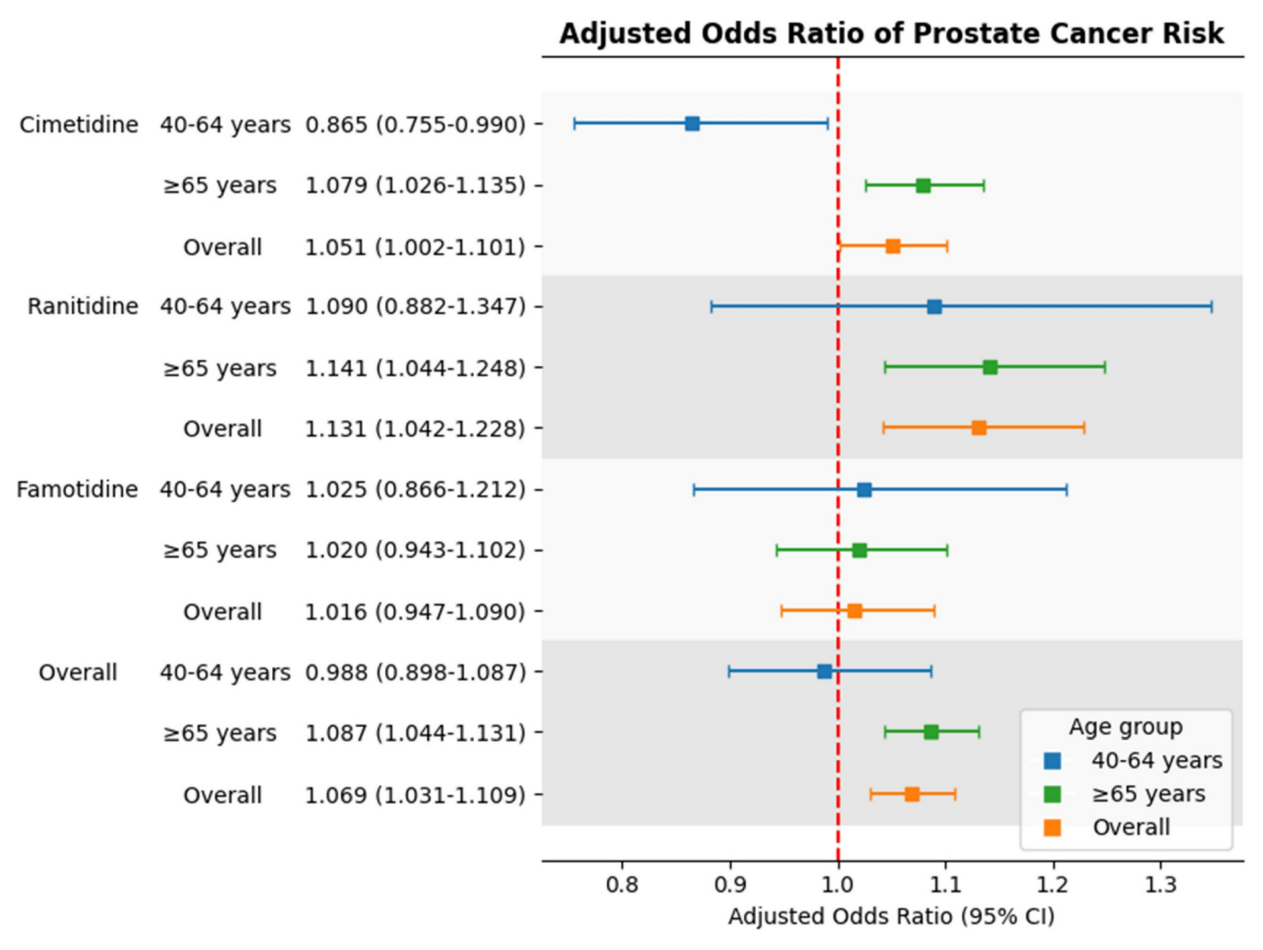
Adjusted odds ratios for prostate cancer risk associated with individual H2 receptor antagonists by age group. Squares represent adjusted odds ratios; horizontal lines indicate 95% confidence intervals. The red dashed line marks adjusted odds ratio = 1.0.

Figure 2 presents a forest plot summarizing the aORs for prostate cancer risk associated with each drug across age groups. The findings show consistent increased risk for cimetidine and ranitidine in older adults, while famotidine use remained unassociated with prostate cancer in all age categories.

## 4. Discussion

This large-scale, population-based case-control study using claims and registry data from Taiwan's HWDC provides evidence that long-term use of H₂RAs is associated with a modest but statistically significant increase in prostate cancer risk, particularly among individuals aged 65 years and older. Stratified analyses revealed that both cimetidine and ranitidine users in this age group exhibited elevated adjusted odds ratios for prostate cancer, whereas famotidine use was not significantly associated with risk across any age category. In contrast, cimetidine use among individuals aged 40-64 years showed an inverse association; however, this finding should be interpreted cautiously given the potential for residual confounding.

Among the H₂RAs examined, cimetidine demonstrated the most distinct age-dependent pattern. The inverse association observed in middle-aged men (40-64 years) is consistent with previous studies suggesting cimetidine's anti-cancer potential 31-34, which may be attributable to its unique endocrine-modulating properties. Unlike other H₂RAs, cimetidine exhibits anti-androgenic activity by competitively inhibiting the binding of dihydrotestosterone and testosterone to androgen receptors, and by interfering with cytochrome P450-mediated sex hormone metabolism 31, 32. Given the critical role of androgen signaling in prostate cancer development 35, partial blockade in individuals with physiologically normal androgen levels may attenuate prostatic stimulation, which might partly explain the observed inverse association.

However, the association between cimetidine use and prostate cancer risk appears to shift with age. Among older men (≥ 65 years), we observed an increased risk, potentially indicating an age-related vulnerability. Although previous studies did not specifically investigate older populations, some evidence has raised concerns about the carcinogenic potential of prolonged or high-dose cimetidine exposure 10, 11. A cohort study reported an elevated risk among men with daily cimetidine use for over 10 years compared to nonusers 10, and animal studies have similarly suggested potential tumor-promoting effects with prolonged exposure 11. The exact mechanisms underlying this shift remain unclear. One possible explanation for this age-related risk reversal involves age-associated hormonal decline. In older adults with naturally reduced testosterone levels 35, additional suppression of androgenic signaling induced by cimetidine may disrupt endocrine homeostasis, potentially promoting tumor progression. Specifically, such hormonal insufficiency may favor the dedifferentiation of prostate cells or the selection of androgen-independent clones, ultimately leading to more aggressive disease 4, 5, 36. Clinical evidence supports this hypothesis: low circulating testosterone and estradiol levels are associated with a higher risk of high-grade prostate cancer 36, and chronic androgen deprivation may promote castration-resistant phenotypes through androgen receptor-related signaling involving receptor overexpression, mutations, ligand-independent activation, and bypass pathways 4, 5.

Ranitidine, in contrast, has been the subject of recent safety concerns due to NDMA contamination 12-14. Although a pharmacovigilance study (n = 21,085) found disproportionality signals linking ranitidine to tumor-related adverse events, particularly in older adults, when compared to all other drugs in the database 37, findings from large population-based cohort studies have not supported an increased cancer risk. Nationwide cohort study from South Korea (n = 88,416) 38, Japanese (n = 113,745) 16, and a multinational cohort study involving 11 international databases 15 all reported no significant association between ranitidine use and prostate cancer risk when compared to other H₂RA users. Despite the lack of association in prior studies, we observed a modest increase in risk among older adults in our cohort. This age-specific association may reflect increased vulnerability to possible ranitidine-related degradation products, including NDMA, although the precise mechanisms remain unclear 12, 39.

Famotidine has been shown to be as effective as cimetidine and ranitidine in the treatment of acid-related disorders, with fewer side effects and no reported anti-androgenic activity 40. Although prior studies compared famotidine with other H₂RAs 16, 41, our findings similarly showed no evidence of an association between famotidine use and prostate cancer risk across any age group when compared to non-users. This supports the possibility that specific drug-related characteristics, rather than H₂RA use as a class, may contribute to the observed associations.

Nonetheless, several limitations should be acknowledged. As with all observational studies, causality cannot be inferred, and residual confounding may persist—particularly from unmeasured lifestyle factors such as smoking, alcohol use, and diet. Prescription records do not guarantee actual medication adherence and do not capture over-the-counter use. We were unable to distinguish mutually exclusive users of individual H₂RAs (e.g., only-cimetidine vs only-ranitidine), as the extracted dataset did not allow reconstruction of exclusive exposure categories, and overlapping exposure may have resulted in exposure misclassification and limited our ability to report mutually exclusive, age-specific exposure counts. Additionally, the ≥ 60-day definition was intended to reflect sustained use, it may not fully capture dose-response relationships. Furthermore, lag-time analysis to exclude exposure immediately before the index date and defined daily dose (DDD)-based dose-response evaluation could not be performed because the extracted dataset did not include segmented exposure windows or DDD information, limiting assessment of potential reverse causation and cumulative dose effects. In addition, specific clinical indications such as gastroesophageal reflux disease or peptic ulcer disease, as well as healthcare utilization measures (e.g., visit frequency or prostate cancer screening) were not available for adjustment. As a result, residual indication and detection bias cannot be fully ruled out. Finally, other H₂RAs such as roxatidine and nizatidine were excluded due to limited or unavailable use in Taiwan, restricting the generalizability of our findings.

In conclusion, our findings underscore the importance of evaluating both class-wide and drug-specific associations in pharmacoepidemiologic research. The differential risk patterns observed among cimetidine, ranitidine, and famotidine users highlight the necessity of considering pharmacologic mechanisms, contamination history, and patient characteristics in interpreting cancer risk signals. Given the widespread historical use of H₂RAs, particularly among older adults, continued monitoring and further drug-specific research are warranted to better inform clinical risk assessment and public health strategies.

## Figures and Tables

**Table 1:** Baseline Characteristics of the Case Group and the Control Group

Characteristics	Cases (*n*=43,578)	Controls (*n* = 174,312)	*p*-value
Age (years), mean ± *SD*	71.18 ± 8.06	71.16 ± 8.01	Matched
20-39, *n* (%)	16 (0.04)	64 (0.04)	Matched
40-64, *n* (%)	9,041 (20.75)	36,164 (20.75)	Matched
≥ 65, *n* (%)	34,521 (79.22)	138,084 (79.22)	Matched
Age-adjusted CCI score, mean ± *SD*	3.58 ± 2.68	3.31 ± 2.57	Matched
Comorbid conditions, *n* (%)			
AIDS/HIV	3 (0.01)	37 (0.02)	.048
Cerebrovascular disease	6,788 (15.61)	28,286 (16.23)	.002
Chronic pulmonary disease	7,445 (17.12)	26,516 (15.21)	< .001
Congestive heart failure	2,140 (4.92)	8,655 (4.97)	.707
Dementia	1,249 (2.87)	6,672 (3.83)	< .001
Diabetes	9,062 (20.84)	37,350 (21.43)	.008
Hemiplegia or paraplegia	216 (0.50)	1,205 (0.69)	< .001
Mild liver disease	4,334 (9.97)	13,996 (8.03)	< .001
Myocardial infarction	567 (1.30)	2,660 (1.53)	< .001
Peptic ulcer disease	10,663 (24.52)	32,527 (18.66)	< .001
Renal disease	3,563 (8.19)	12,166 (6.98)	< .001
Rheumatic disease	460 (1.06)	1,548 (0.89)	< .001
Serious liver disease	24 (0.06)	194 (0.11)	< .001
Concomitant drugs, *n* (%)			
Aspirin	10,964 (25.16)	42,509 (24.39)	< .001
Metformin	8,066 (18.51)	29,671 (17.02)	< .001
Statin	6,230 (14.30)	27,782 (15.94)	< .001

SD, standard deviation

**Table 2:** The association of H2 receptor antagonists with the risk of prostate cancer by age groups.

	Age	40-64 years (n = 45,205)		≥ 65 years (n = 172,605)		Total† (n = 217,890)	
Medication		aOR	95%CI	aOR	95%CI	aOR	95%CI
Overall		0.988	(0.898, 1.087)	1.087***	(1.044, 1.131)	1.069***	(1.031, 1.109)
Cimetidine		0.865*	(0.755, 0.990)	1.079**	(1.026, 1.135)	1.051*	(1.002, 1.101)
Ranitidine		1.090	(0.882, 1.347)	1.141**	(1.044, 1.248)	1.131**	(1.042, 1.228)
Famotidine		1.025	(0.866, 1.212)	1.020	(0.943, 1.102)	1.016	(0.947, 1.090)

* *p* < .05; ** *p* < .01; *** *p* < .001.; † The "Total" category includes all participants aged ≥ 20 years, including those aged 20-39 years (n = 80); aOR, adjusted odds ratio.

## Abbreviations

aOR: adjusted odds ratio
ATC: Anatomical Therapeutic Chemical
CCI: Charlson comorbidity index
CI: confidence interval
HWDC: Health and Welfare Data Science
H₂RA: Histamine-2 receptor antagonist
IARC: International Agency for Research on Cancer
NDMA: N-nitrosodimethylamine
OR: odds ratio
TNM: tumor-node-metastasis
